# Abstracts of the 2021 Canadian Association of Medical Oncologists Annual Meeting

**DOI:** 10.3390/curroncol28030204

**Published:** 2021-06-15

**Authors:** Jonathan Loree, Erin Powell, Sharlene Gill, Stephen Welch, Bruce Colwell, Desiree Hao

**Affiliations:** Canadian Association of Medical Oncologists, Ottawa, ON K1E 3R9, Canada

**Keywords:** medical oncology, cancer, research

## Abstract

On behalf of the Canadian Association of Medical Oncologists, we are pleased to present the Abstracts of the 2021 Annual Meeting. The National CAMO Residents Research Day was held virtually on 1 April 2021 and the CAMO Virtual Annual Scientific Meeting (ASM) & Annual General Meeting (AGM) took place on 22 April 2021. Twenty (20) abstracts were selected for presentation as oral presentations and rapid-fire presentations. Awards for the top three (3) abstracts were presented during the ASM and AGM. All of them were marked as “Award Recipient”. We congratulate all the presenters on their research work and contribution.

*01_CAMO_2021 (Award Recipient)*

**Barriers to Access of Contemporary Treatment for Lethal Prostate Cancer: An Ontario Population-Based Study**

## 

LeighJennifer^1^*QureshiDanial^2^SuchaEwa^3^WebberColleen^2^TanuseputroPeter^2^,^3^OngMichael^4^1Department of Medicine, University of Ottawa, Ottawa, ON K1N 6N5, Canada2Ottawa Hospital Research Institute, Ottawa, ON K1H 8L6, Canada3ICES University of Ottawa, Ottawa Hospital Research Institute, Ottawa, ON K1H 8L6, Canada4Department of Medicine, the Ottawa Hospital Cancer Center, Ottawa, ON K1H 8L6, Canada*Correspondence: jleigh@toh.ca

 

## 1. Objective

Our objective was to investigate and describe the factors important to receipt of novel life prolonging therapy (LPT) in patients with lethal castration-resistant prostate cancer in Ontario.

## 2. Methods

Population-based administrative databases from Ontario (Canada) were used to identify patients 65 years or older with prostate cancer who were eligible for Ontario Drug Benefit 2002–2018 (*n* = 138,976), received continuous androgen deprivation therapy (ADT, *n* = 37,578), and died of prostate cancer-specific death between 2013–2017 (*n* = 3575). Baseline and treatment characteristics were analyzed for association with receipt of therapy in a 2-year observation period prior to death.

## 3. Results

Only 40.4% were identified to receive LPT in the two years preceding death despite 51.3% presenting with metastasis. Type of LPT received included abiraterone (66.3%), docetaxel (50.3%), enzalutamide (17.2%), radium-223 (10.0%), and cabazitaxel (3.5%). LPT access increased with cancer centre consultation (yes: 50.2%; no: 22.5%, *p* < 0.0001), and type of oncologist involved (urologist: 39.1%; radiation: 48.3%; medical: 56.5%, *p* < 0.0001). Accessibility decreased with advanced age (65–74 y: 58.8%; 75–84 y: 41.3%; 85+ y: 22.3%, *p* < 0.0001), greater number of chronic conditions (0: 49.4%, 1–4: 41.6%, 5+: 29.2%, *p* < 0.0001), and long-term care registration (yes: 7.8%; no: 41.2%, *p* < 0.0001). Proportion of patients receiving LPT within two years of death significantly increased with decedent year (2013: 22.7%, 2014: 31.8%, 2015: 41.8%, 2016: 49.1%, 2017: 57.9%). LPT receipt was not associated with income quartile, rurality index, patient distance to cancer centre, or metastatic status at diagnosis.

## 4. Conclusions

A high proportion of patients dying of prostate cancer in Ontario never receive LPT, although large increases in LPT by year and new indications for LPT use are poised to address the shortfall. Our provincial health care system did not discriminate on the basis of income, remoteness or rurality for access to LPT. Improving access to cancer centre consultation may be important to further improve delivery.

## 

*02_CAMO_2021*

**A Review of OBSP Screening Trends Across the Province—A Retrospective Population-Based Study Using Administrative Data**

## 

FebbraroMichela*PondGregoryBellKathleenKumar-TyagiNidhiDhesy-ThindSukhbinderJuravinski Cancer Center, Hamilton, ON L8V 5C2, Canada*Correspondence: michela.febbraro@medportal.ca

 

## 1. Background

The Ontario Breast Screening Program (OBSP) was introduced to provide high-quality breast cancer screening services. Despite universal access to OBSP services, screening and diagnosis rates differ across the province. This study sought to identify whether patient factors act synergistically with regional aspects of care and impact OBSP screening. 

## 2. Methods

A retrospective population-based study using linked administrative health care data through ICES (formally the Institute for Clinical Evaluative Sciences) was conducted between 2009 and 2016. The study cohort was defined as all screen eligible women (aged 51–74 years) with breast cancer living in Ontario (Canada). The primary outcome was OBSP screening within 730 days prior to diagnosis. Prognostic factors for OBSP use in the screened cohort were identified using logistic regression. 

## 3. Results

Screen-eligible women (44,732) were diagnosed with breast cancer with 17,800 (39.8%) receiving OBSP screening within 730 days prior to breast cancer diagnosis. 35,844 women (80%) were diagnosed with stage I/II breast cancer. Of these, 43.7% had OBSP screening within 730 days prior to diagnosis. In contrast, 6878 women had stage III/IV breast cancer, of whom, 25.5% had prior OBSP screening (chi-square *p*-value < 0.001). In multivariable model, increasing age (odds ratio [OR] 1.29, 95% confidence interval [CI] 1.27–1.31) and rural LHIN location (OR 1.14, 95% CI 0.96–1.36) were more likely to receive OBSP screening. Charlson score (2+ vs. 0–1, OR 0.58, 95% CI 0.55–0.60), previous cancer (OR 0.87, 95% CI 0.78–0.98), and higher marginalization index (OR 0.95, 95% CI 0.93–0.96) were less likely to have OBSP screening prior to diagnosis. 

## 4. Conclusions

OBSP screening is associated with lower stage breast cancer. However, regional variations in OBSP screening are dependent upon several factors, the most important being population density and marginalization which need to be addressed independently in order to overcome barriers to care. 

## 

*03_CAMO_2021*

**Beyond BRCA? Clinical Utility of Homologous Recombination Deficiency in Gastrointestinal Cancers**

## 

TsangErica S.^1^*CsizmokVeronika^2^WilliamsonLaura M.^2^PleasanceErin^2^TophamJames^3^KarasinskaJoanna^3^TitmussEmma^2^SchraderIntan^2^CaffertyFergus^4^YipStephen^5^Tessier-CloutierBasile^2^MungallKaren^2^NgTony^5^SunSophie^1^LimHoward J.^1^LoreeJonathan M.^1^LaskinJanessa^1^MarraMarco^2^,^6^JonesSteven^2^,^6^,^7^SchaefferDavid F.^3^,^5^RenoufDaniel J.^1^1Department of Medical Oncology, BC Cancer, Vancouver, BC V5Z 4E6, Canada2Canada’s Michael Smith Genome Sciences Center at BC Cancer, Vancouver, BC V5Z 4S6, Canada3Pancreas Center BC, Vancouver, BC V5Z 1L8, Canada4Department of Radiology, BC Cancer, Vancouver, BC V5Z 4E6, Canada5Department of Pathology and Laboratory Medicine, University of British Columbia, Vancouver, BC V5Z 1M9, Canada6Department of Medical Genetics, University of British Columbia, Vancouver, BC V6H 3N1, Canada7Department of Molecular Biology, Simon Fraser University, Burnaby, BC V5A 1S6, Canada*Correspondence: erica.tsang@bccancer.bc.ca

 

## 1. Background

There is emerging evidence about the predictive role of homologous recombination deficiency (HRD), but the clinical utility is less well defined in gastrointestinal (GI) malignancies. 

## 2. Methods

We reviewed the whole genome (WGS) and transcriptomic (RNA-Seq) data of patients with advanced GI cancers between 2012–2018 in the Personalized Oncogenomics trial (NCT02155621). HRD was defined as a score ≥34, and a high mutational signature 3 score was defined as >0.05. Retrospective chart review was conducted to extract treatment and survival outcomes. Overall survival (OS) from initiation of first-line systemic therapy and time to progression on platinum therapy (TTPp) were calculated. Linear and multivariable regression analyses were conducted. 

## 3. Results

Of 154 patients with GI primaries, 56% were male and 105 (68%) were exposed to a platinum agent in the metastatic setting. Primary sites included upper GI (*n* = 20, 9%), pancreas (*n* = 35, 16%), colorectal (*n* = 74, 33%), and other GI primary (*n* = 25, 11%). Ten patients (6%) had a *BRCA1/2* mutation, 20 (13%) had a high HRD score, and 11 (7%) had a high signature 3 score (>0.05). Six patients had both high HRD and high signature 3 scores.

On linear regression, high HRD scores and mutational signature 3 were independently associated with longer TTPp (β = 4.17, 95% CI 0.15–8.19, *p* = 0.04; β = 8.03, 95% CI 2.87–13.18, *p* < 0.05, respectively). On multivariable linear regression, after adjusting for HRD score, *BRCA1/2* status, and tumor site, only cases with a mutational signature 3 retained significance (*p* < 0.05). HRD status was not prognostic for OS (HR 1.02, 95% CI 0.65–1.62, *p* = 0.92). 

## 4. Conclusions

Within a cohort of patients with GI malignancies, mutational signature 3 was more strongly associated with TTPp compared to HRD score. These data highlight potential predictive implications of Signature 3 to complement HRD and *BRCA* status in identifying patients who may benefit from exposure to platinum therapy. 

## 

*04_CAMO_2021*

**Dosing, Effectiveness and Safety of Lenvatinib in the Real-World Treatment of Hepatocellular Carcinoma: Results from a Canadian Multicenter Database (HCC CHORD)**

## 

AmaroCarla P.^1^*AllenMichael J.^2^KnoxJennifer J.^2^TsangErica S.^3^LimHoward J.^3^Lee-YingRichard M.^1^ChanKelvin K. W.^4^QianJessica^5^MeyersBrandon M.^5^ThawerAlia^4^Al-SaadiSulaiman M. S.^6^HsuTina^6^RamjeesinghRavi^7^KarachiwalaHatim^8^AbedinTasnima^1^TamVincent C.^1^on behalf of the HCC CHORD Consortium1Tom Baker Cancer Centre, University of Calgary, Calgary, AB T2N 1N4, Canada2Princess Margaret Cancer Centre, University of Toronto, Toronto, ON M5S 1A8, Canada3BC Cancer, Vancouver, BC, Canada4Sunnybrook Odette Cancer Centre, University of Toronto, Toronto, ON M5S 1A8, Canada5Juravinski Cancer Centre, Hamilton, ON L8V 5C2, Canada6Ottawa Regional Cancer Centre, Ottawa, ON K1G 3Y9, Canada7Nova Scotia Cancer Centre, Dalhousie University, Nova Scotia, NS B3H 4R2, Canada8Cross Cancer Institute, Edmonton, AB T6G 1Z2, Canada*Correspondence: amarocal@hotmail.com

 

## 1. Objective

To assess the real-world effectiveness and safety of lenvatinib in advanced hepatocellular carcinoma (HCC).

## 2. Methods

From July 2018 to December 2019, HCC patients treated with lenvatinib from 10 different Canadian cancer centers were included. Overall survival (OS), progression-free survival (PFS) and objective response rate (ORR) were retrospectively analyzed and compared across first- and 2nd/3rd-line use of lenvatinib. In first-line patients, OS was also compared between different mean dose-intensities and starting dose groups. 

## 3. Results

A total of 220 patients were included. Median follow-up was 4.5 months. A total of 79% patients received lenvatinib as first-line therapy. ORR, PFS and OS results and their comparison between the different lines of therapy are shown in the table. Considering the patients who received lenvatinib first-line, 40% received a mean dose intensity of 67% or less. Median OS for mean dose intensity >67% and ≤67% were 13.7 and 7.7 months (*p* = 0.009), respectively. Of these first-line patients, 54% started lenvatinib at full dose according to their weight. Median OS for starting lenvatinib at full and reduced dose was 12.3 and 15.8 months (*p* = 0.75), respectively. Toxicities occurred in 86% of patients and led to drug discontinuation in 24% patients. The most common side effects were fatigue (59%) and hypertension (41%).

## 4. Conclusions

Lenvatinib appears to be effective and safe in real-world practice regardless of the line of therapy, with results in first-line comparable to those demonstrated in the REFLECT trial. For patients who received lenvatinib first-line, treatment mean dose-intensity of >67% may improve survival while starting dose does not appear to affect survival.

## 

*05_CAMO_2021*

**The Influence of Adjuvant Chemotherapy Dose Intensity on Overall Survival in Resected Colon Cancer: A Multicentre Retrospective Analysis**

## 

LakkunarajahSuganija^1^*BreadnerDaniel^2^ZhangHanbo^3^KongShiying^4^CheungWinson^4^LoreeJonathan^5^WelchStephen^2^1Department of Medicine, Schulich School of Medicine, London, ON N6A 5C1, Canada2Department of Oncology, Schulich School of Medicine, London, ON N6A 5C1, Canada3Department of Oncology, Cross Cancer Institute, Edmonton, AB T6G 1Z2, Canada4Department of Oncology, Arnie Charbonneau Cancer Institute, Calgary, AB T2N 4Z6, Canada5Department of Oncology, British Columbia Cancer Agency, Vancouver, BC V5Z 1L3, Canada*Correspondence: suganija.lakkunarajah@lhsc.on.ca

 

## 1. Background

Colorectal cancer remains the second leading cause of cancer death in developed countries, despite the implementation of early detection and screening programs. There are many notable trials showing the benefit of using fluorouracil-based chemotherapy in the addition of oxaliplatin such as modified fluorouracil (5-FU), leucovorin and oxaliplatin (FOLFOX) and capecitabine and oxaliplatin (CAPOX). There is evidence that achieving a 5-FU dose intensity (DI) > 70–80% in adjuvant colon cancer treatment improves overall survival (OS). The oxaliplatin dose intensity threshold under which survival is inferior is not established. 

## 2. Methods

Patients treated with adjuvant chemotherapy between 2006 and 2011 for resected stage III colon cancer (CC) from four academic cancer centres in Canada were retrospectively analysed. Patients that received CAPOX and FOLFOX were examined for the relationship between DI and OS. 

## 3. Results

625 patients were analysed with resected high risk stage II or stage III CC that received adjuvant chemotherapy. The median age was 63. 34.3% and 31.5% patients had T4 and N2 disease, respectively. Median follow was 38.2 months. The median oxaliplatin DI was 70%. 56.8% of patients had an oxaliplatin DI of >80%. An oxaliplatin DI of >80% was associated with a significant improvement in survival, HR = 0.45 (95% CI 0.24–0.86, *p* < 0.01). Achieving a DI of >80% for capecitabine or 5-FU did not improve OS. Other factors associated with inferior OS included T4 (HR = 2.9, *p* = 0.05) and N2 (HR = 5.15, *p* = 0.0007) subgroups. 

## 4. Conclusions

Patients receiving adjuvant chemotherapy with an oxaliplatin DI of >80% for high risk stage II and stage III CC have a superior OS. 

## 

*06_CAMO_2021 (Award Recipient)*

**Impact of TAILORx Data on Chemotherapy Prescribing in British Columbia**

## 

TeschMegan^1^*SpeersCaroline^2^DioceeRekha^2^NicholAlan^3^LohrischCaroline^1^1Department of Medical Oncology, BC Cancer, Vancouver, BC V8R 6V5, Canada2Breast Cancer Outcomes Unit, BC Cancer, Vancouver, BC V8R 6V5, Canada3Department of Radiation Oncology, BC Cancer, Vancouver, BC V8R 6V5, Canada*Correspondence: megan.tesch@bccancer.bc.ca

 

## 1. Background

The 21-gene recurrence score assay (RS) reduces adjuvant chemotherapy use in hormone+, HER2-, node-breast cancer, justifying the assay’s cost. The TAILORx trial confirmed the predictive value of RS and established thresholds for chemotherapy benefit in younger and older patients. We examined chemotherapy use in BC post-TAILORx publication, as a prelude to exploring age-adjusted cost-effectiveness of the assay.

## 2. Methods

We assembled three cohorts of patients with hormone+, HER2-, node- breast cancer: diagnosed before RS funding (cohort 1: 1 January 2013–31 December 2013), after public funding (cohort 2: 1 July 2015–30 June 2016), and post-TAILORx (cohort 3: 1 July 2018–30 June 2019). Patients 18–80 years old (yo) with tumors that were grade 3, grade 2 ≥ T1b, or any T size and grade if ≤40 yo were included, matching BC funding criteria. Chemotherapy use was compared between cohorts using univariate analyses.

## 3. Results

2066 patients met inclusion criteria. Chemotherapy use in cohorts 1, 2, and 3 was 21%, 17%, and 13%, respectively. Chemotherapy use declined by 19% after RS funding and by another 23% post-TAILORx (*p* = 0.001). Reduction in chemotherapy use was significant for RS 11–20 tumors (cohort 3 vs. 2, *p* = 0.004). A 7.5% nonsignificant increase in chemotherapy use was seen for RS 26–30 tumors (cohort 2 vs. 3, *p* = 0.55). There was no significant change in chemotherapy use in patients > 50 yo (12% in cohort 2 vs. 10% in cohort 3, *p* = 0.22). Among patients 70–80 yo in cohort 3 with RS, 14% had RS ≥ 26, and of these, 40% had chemotherapy, compared with 92% chemotherapy use for patients ≤50 yo with RS ≥ 26.

## 4. Conclusions

Chemotherapy use decreased post-TAILORx, driven primarily by RS 11–20 tumors and patients ≤50 yo. Chemotherapy use changed little in patients >50 yo, suggesting trial results confirmed pre-existing prescribing practices, and increased for RS 26–30 tumors, reflecting acceptance of TAILORx thresholds for chemotherapy benefit. Chemotherapy use was low overall in patients >50 yo, especially in those 70–80 yo, in part due to the low frequency of high RS tumors. Given these findings, we conclude that cost-effectiveness modelling for publicly funded RS should take age into consideration. 

## 

*07_CAMO_2021*

**Optimizing Cabazitaxel (Cbz) vs. Novel Anti-Androgens (NAA) Abiraterone (Abi) or Enzalutamide (Enz) Post-Docetaxel (Dtx) in Metastatic Castrate Resistant Prostate Cancer (mCRPC)**

## 

WatsonAlexander S.*GagnonRichardBatuyongEugeneAlimohamedNimiraLee-YingRichardTom Baker Cancer Centre, University of Calgary, Calgary, AB T2N 4N2, Canada*Correspondence: alexander.watson@albertahealthservices.ca

 

## 1. Objective

Treatment sequencing post-Dtx in mCRPC remains uncertain, with Cbz chemotherapy anecdotally underutilized. The recent CARD trial suggested Cbz may have benefit over NAA in patients who had rapid progression within 12 months (RP) on a previous NAA. We sought to characterize real-world Cbz use and factors interacting with clinical outcomes. 

## 2. Methods

mCRPC patients in Alberta who received Dtx from October 2012 to 31st December 2017 were assessed. We examined Cbz eligibility per trial criteria, tracked therapies, outcomes, and documented therapy discussions. OS was measured using the Kaplan-Meier method and compared via log-rank test. Agent utilization and outcome interactions were analysed via Chi-Square.

## 3. Results

593 mCRPC patients received Dtx over the study period. 338 patients (57%) were Cbz-eligible per TROPIC trial criteria, with ineligibility most often for Dtx intolerance (14%) or comorbidities (14%). Patients with RP on first NAA had poorer OS (12.3 vs. 24.8 months, *p* < 0.001). OS was increased among RP patients who received Cbz (16.9 vs. 10.3 months, *p* = 0.015), but not improved without RP (17.1 vs. 32 months, *p* = 0.084). The most common agents post-Dtx were Abi (280, 47%) and Enz (250, 42%). Significantly fewer patients (177, 30%) received Cbz (*p* < 0.001). Immediately post-Dtx, 398 patients (67%) did not have a documented discussion around Cbz, and in 238 cases (40%) Cbz consideration was never documented. Patient choice against Cbz was recorded in 16% of discussions.

## 4. Conclusions

In a real-world mCRPC cohort, Cbz was less utilized then NAA post-Dtx. Provider preference was a major factor, with Cbz discussions limited post-Dtx, despite many patients being eligible. Cbz use was associated with improved OS among patients who had RP on first NAA, a subset with worse outcomes overall. These real-world data suggest Cbz use could be optimized by focusing on patients with RP on prior NAA. 

## 

*08_CAMO_2021*

**Real World Outcomes of Metastatic Breast Cancer (MBC) Patients with Brain Metastases (BrM) Treated with Radiotherapy in Ontario: A Population-Based Study**

## 

WangXin Y.^1^*ChehadeRania^1^,^2^SahgalArjun^1^,^2^SolimanHany^1^,^2^RosenMichael^3^SaskinRefik^4^ZhangBo^4^ChanKelvin K. W.^1^,^2^JerzakKatarzyna J.^1^,^2^1Department of Medicine, University of Toronto, Toronto, ON M4N 3M5, Canada2Division of Medical Oncology, Sunnybrook Health Sciences Centre, Toronto, ON M4N 3M5, Canada3Faculty of Medicine, University of Ottawa, Ottawa, ON K1N 6N5, Canada4Institute for Clinical Evaluative Sciences (ICES), Sunnybrook Health Sciences Centre, Toronto, ON M4N 3M5, Canada*Correspondence: stevenxy.wang@mail.utoronto.ca

 

## 1. Objectives

Identify treatment patterns and outcomes among women treated with radiotherapy for breast cancer BrM in Ontario.

## 2. Methods

We used the Ontario-wide ICES database to identify patients diagnosed with de-novo MBC between January 2009 and December 2018. Primary endpoints included were (i) cumulative incidence of radiotherapy for BrM accounting for the competing risk of death (calculated using the Cumulative Incidence Function), and (ii) time from MBC diagnosis to treatment with brain radiotherapy. The key secondary endpoint was overall survival (OS). Kaplan-Meier analyses were performed for time-to-event endpoints. Univariable and multivariable logistic regression analyses were used to account for potential confounding variables. Data were censored if patients were alive at last available follow-up with the last cut-off date being 31 March 2019. 

## 3. Results

3916 patients with de-novo MBC were identified, among whom 549 (14%) developed BrM requiring radiotherapy; patients with HER2+ (23.0%) and triple negative breast cancer (TNBC) (20.9%) were most likely to require brain radiotherapy.

The median time from MBC diagnosis to treatment for BrM was 15 months, ranging from 7.5 months to 19.8 months for patients with TNBC and HER2+/HR+ MBC, respectively.

The median OS from the time of brain radiotherapy was 5.1 months, ranging from 2.6 months to 9.4 months for TNBC and HER2+/HR− populations, respectively. In a multivariable Cox regression model, HER2-negative status, treatment with whole brain radiotherapy (WBRT), lower income quintile, and age >60 were independently prognostic for shorter OS. Patients treated with stereotactic radiosurgery (SRS) had lower 30-day mortality (6.4% vs. 18.9%, *p* = 0.003) and lower likelihood of hospitalization (9.6% vs. 20.2%, *p* = 0.015) compared to patients treated with WBRT.

## 4. Conclusions

Approximately one in seven patients with MBC in Ontario will require radiotherapy for BrM. Our data support the use of SRS when indicated and provide insights regarding the time to development of BrM by breast cancer subtype.

## 

*09_CAMO_2021*

**Examination of Febrile Neutropenia and the Utilization of G-CSF on Healthcare Systems**

## 

PenczAlec^1^BlackMorgan^1^StrumScott^2^GipsonMeghan^3^VincentMark^1^*1Department of Oncology, London Regional Cancer Program, London, ON N6A 5W9, Canada2Department of Medicine, Schulich School of Medicine and Dentistry, London, ON N6A 5C1, Canada3Department of Medicine, Royal College of Surgeons in Ireland, Dublin D02 YN77, Ireland*Correspondence: mark.vincent@lhsc.on.ca

 

## 1. Objective

Febrile neutropenia (FN), a hallmark toxicity of myelosuppressive chemotherapy, frequently results in chemotherapy delays, dose decreases, or premature cessation. The utilization of granulocyte colony-stimulating factor (G-CSF) has been prescribed to mitigate this problem and has been widely studied. The biosimilar Grastofil^®^ (a form of G-CSF, Apotex, Toronto ON, Canada), provincially funded in December 2016, is a less expensive but high-quality alternative to Neupogen^®^ (Amgen, Thousand Oaks, CA, USA) Increasing availability of G-CSF to patients might decrease hospitalizations and costs. 

## 2. Methods

A retrospective chart review was carried out on 158 patients treated at the London Regional Cancer Program (from 1 September 2015–30 June 2018) with non-haematological, solid tumors, whom experienced FN. Patients were arranged into two cohorts: before (1 September 2015–30 November 2016), and after (1 April 2017–30 June 2018), Grastofil^®^ funding. Comparative analyses were completed and Student’s T was calculated to determine statistical significance. 

## 3. Results

After the introduction of Grastofil^®^, the frequency of FN in all patients with cancer decreased by 29.85% (*p* = 0.0190, pre FN-Rate 7.70%, post FN-rate 5.40%), and the length of hospital stay for each FN patient decreased by 25.87% (*p* = 0.105, Pre: 11.40 days/pt. Post: 8.45 days/pt.). Furthermore, the absolute number of FN patients who received G-CSF support as primary prophylaxis increased by 90.91% (*p* = 0.0714). Finally, comparison of average costs revealed a savings of $42,117.78 for every 200 patients started on cytotoxic chemotherapy (*p* = 0.0116). This data was consistent with the hypothesis that increased availability and usage of G-CSF led to a decrease in FN admissions, length of stay, and costs.

## 

*10_CAMO_2021*

**Current Attitudes toward Unfunded Cancer Therapies among Canadian Medical Oncologists**

## 

WongSelina K.*GondaraLovedeepGillSharleneBC Cancer, Vancouver, BC V5Z 4E6, Canada*Correspondence: selina.wong1@bccancer.bc.ca

 

## 1. Objective

To describe the frequency and predictors of discussion of unfunded cancer treatments among Canadian medical oncologists.

## 2. Methods

A REDCap survey with multiple choice and case-based scenarios was distributed in July 2020. Descriptive statistics and multivariate logistic regression were performed.

## 3. Results

116 responses were received: BC (35%), ON (27%) and AB (11%), 53% female, 88% from a comprehensive cancer center (CCC), and 47% in practice for >15 years. 

48% reported that they would discuss unfunded treatments if recommended in guidelines even if not Health Canada (HC) approved, while 50% would only do so for HC approved treatments. Only 2% of respondents would never discuss unfunded treatments. Per Table 1, respondents in practice >15 years were significantly less likely to discuss treatments that are not HC approved compared to those in practice <5 years: OR 0.14, *p* = 0.002. Other variables were not statistically significant.

Main predictors of increased likelihood of discussing unfunded treatments: availability of a manufacturer compassionate access or co-pay program, patient willingness to pursue self-pay, and if the patient has private insurance. 90% indicated moderate to extreme levels of concern regarding the future of Canadian cancer drug funding.

## 4. Conclusions

Given fiscal limitations within our publicly funded system, an increasing proportion of cancer therapies may not receive approval for public funding. Our survey reveals variability in practice with respect to discussing unfunded therapies, with years in practice as a significant determinant of willingness to discuss. 

## 

*11_CAMO_2021*

**Prognostic Pathological and Clinical Factors Associated with Overall Survival in Metastatic Melanoma Undergoing Anti PD-1 Treatment**

## 

KoczkaKim^1^*RigoRodrigo^1^BatuyongEugene^2^AsadMohammed^2^SuoAleksi^1^WangEdwin^2^ChengTina^1^1Department of Oncology, University of Calgary, Calgary, AB T2N 1N4, Canada2Cumming School of Medicine, University of Calgary, Calgary, AB T2N 1N4, Canada*Correspondence: kim.koczka@albertahealthservices.ca

 

## 1. Background

Anti-PD-1 immunotherapy has revolutionized metastatic melanoma treatment as first-line monotherapy or in combination with Ipilimumab. Unfortunately, 30–50% of patients will progress within 3 months regardless of treatment, with limited evidence on who derives benefit from immunotherapy. We report clinical and histological predictive and prognostic factors from a multi-institutional cohort. 

## 2. Methods

Patients between 2014–2017 treated with Nivolumab and Pembrolizumab monotherapy were identified from a provincial pharmacy database in Alberta, Canada. All patients had stage IV melanoma. Patient characteristics, investigations, treatment and clinical outcomes were obtained from electronic medical records. We utilized Cox regression and Kaplan-Meier methods to analyze median progression free survival (mPFS) and median overall survival (mOS).

## 3. Results

143 patients with either cutaneous (114) or melanoma of unknown primary (29) were identified. Immunotherapy was median second line treatment and patients received a median of 7 doses. The overall response rate was 33% with a median follow up time of 37.1 months. Ulcerated primary tumors had a worse mOS of 11.8 months vs. 19.3 months (*p* = 0.042). Other histological factors (including Breslow, tumor infiltrating leukocytes, mitosis) were not associated with PFS or OS. Clinical factors associated with worsened PFS and OS were liver metastases, and ≥3 sites of disease. Elevated LDH, platelets, neutrophils, and lower hemoglobin, lymphocytes, and a neutrophil/lymphocytes ratio were associated with worse PFS and OS. We identified 4 prognostic subgroups using LDH and number of visceral metastases (Table 1) which was statistically significant for PFS and OS.

## 4. Conclusions

Ulcerated primary tumors, liver metastasis, and more sites of disease had worse PFS and OS. We identified prognostic clinical factors associated with PFS and OS, along with 4 subgroups of patients.

## 

*12_CAMO_2021*

**Efficacy of Perioperative Chemotherapy in Resected Colorectal Liver Metastases: A Systematic Review and Meta-Analysis**

## 

BosmaNicholas A.^1^,^2^*KeehnAlysha R.^3^,^4^Lee-YingRichard^1^,^2^MacLeanAnthony R.^3^BrennerDarren M.^2^,^4^1Department of Medicine, University of Calgary, Calgary, AB T2N 1N4, Canada2Department of Oncology, University of Calgary, Calgary, AB T2N 1N4, Canada3Department of Surgery, University of Calgary, Calgary, AB T2N 1N4, Canada4Department of Community Health Sciences, University of Calgary, Calgary, AB T2N 1N4, Canada*Correspondence: nicholas.bosma@ahs.ca

 

## 1. Background

Nearly half of patients with colorectal cancer develop liver metastases. Radical resection of colorectal liver metastases (CRLM) offers the best chance of cure, significantly improving 5-year survival. Recurrence of metastatic disease is common, occurring in 60% or more of patients. Clinical equipoise exists regarding the role of perioperative chemotherapy in patients with resected CRLM and the optimal regimen and sequencing of chemotherapy remain to be elucidated for this population.

## 2. Objective

To investigate the efficacy of perioperative chemotherapy in patients that have undergone curative-intent resection of CRLM. 

## 3. Methods

A systematic review and meta-analysis was completed of randomized controlled trials (RCTs) comparing perioperative chemotherapy to surgery alone in patients with resected CRLM. MEDLINE (Ovid), EMBASE and Cochrane Central Register of Controlled Trials (CENTRAL) databases were searched as well as abstracts published within the last 5 years from the American Society of Clinical Oncology (ASCO) and the European Society for Medical Oncology (ESMO) conferences. A meta-analysis was performed pooling the hazard ratios for disease-free survival (DFS) and overall survival (OS), using a random-effects model. 

## 4. Results

A total of five, phase 3, open-label RCTs were included, resulting in a pooled analysis of 1119 of the total 1146 enrolled patients. 559 patients were randomized to perioperative chemotherapy and 560 to surgery alone. Pooled estimates demonstrated a statistically significant improvement in DFS (HR 0.71, 95% CI: 0.61–0.82; *p* < 0.001) but not OS (HR 0.87, 95% CI: 0.73–1.04; *p* = 0.136). 

## 5. Conclusions

Perioperative chemotherapy in the setting of resected CRLM was associated with an improvement in DFS, however this did not translate into an OS benefit. Poor compliance to post-hepatectomy oxaliplatin-based chemotherapy regimens was identified. Further investigation into the optimal regimen and sequencing of perioperative chemotherapy is justified. 

## 

*13_CAMO_2021*

**Efficacy and Toxicity of Combined Inhibition of EGFR and VEGFR in Advanced Non-Small-Cell Lung Cancer Patients Harboring Activating EGFR Mutations: A Systematic Review and Meta-Analysis**

## 

DeluceJasna^1^,^2^,^3^*MajDavid^1^,^3^BoldtGabriel^2^,^3^BreadnerDaniel^1^,^2^,^3^RaphealJacques^1^,^2^,^3^1Schulich School of Medicine and Dentistry, London, ON N6A 5C1, Canada2London Regional Cancer Program, Division of Medical Oncology, London, ON N6A 5W9, Canada3London Health Sciences Centre, London, London, ON N6A 5W9, Canada*Correspondence: jasna.deluce@lhsc.on.ca

 

## 1. Background

Dual inhibition of epidermal growth factor receptor (EGFR) and vascular endothelial growth factor (VEGF) pathways have demonstrated promising results for treatment of advanced non-small cell lung cancer (NSCLC). We conduced a systematic review and meta-analysis to assess the efficacy and toxicity of the combined treatment with EGFR tyrosine kinase inhibitors (TKIs) and VEGF monoclonal antibodies (MABs) for advanced NSCLC patients harboring activating EGFR mutations, in comparison EGFR TKIs alone.

## 2. Methods

The electronic databases PubMed, Cochrane and EMBASE, were searched for relevant randomized trials between 2000 and 2019. The primary endpoints were overall survival (OS) and progression-free survival (PFS). Secondary endpoints included objective response rate (ORR), disease control rate (DCR), and grade 3 or higher adverse events (AEs). Pooled hazard ratios (HR) for OS and PFS and odds ratios (OR) for ORR, DCR and toxicity were meta-analyzed using the generic inverse variance and the Mantel-Haenszel methods. Random-effect models were used to compute pooled estimates. Subgroup analyses compared PFS by gender, age, smoking status, type of EGFR mutation, intra-cranial disease and ECOG status. 

## 3. Results

A total of 1246 patients from six trials were evaluated for analyses. The combination treatment decreased the risk of disease progression (PFS) (HR = 0.64; 95% CI, 0.55–0.75), but had no effect on OS compared to EGFR inhibition alone (HR = 0.90; 95% CI, 0.68–1.19). There was a significantly increased number of AEs reported in the dual treatment arm (OR = 3.55; 95% CI 2.74–4.59), with proteinuria (OR = 14.55; 95% CI 4.47–47.4) and hypertension (OR = 7.02; 95% CI 4.73–10.43) being the most significantly increased AEs. Furthermore, no difference in ORR and DCR was found. The PFS benefit was consistent across all subgroups. 

## 4. Conclusions

This study suggests combined inhibition of EGFR and VEGF pathways significantly improves PFS, with no interim OS benefit, and increases AEs. Mature OS data are needed to strengthen these results along with results from newer trials exploring this strategy with 3rd generation EGFR-TKIs.

## 

*14_CAMO_2021*

**PD-L1 Expression in Breast Cancer Brain Metastases**

## 

ChehadeRania^1^*QaziMaleeha A.^2^Nofech-MozesSharon^3^JerzakKatarzyna J.^4^1Department of Medical Oncology, Faculty of Medicine, University of Toronto, Toronto, ON M5S 1A1, Canada2Faculty of Medicine, University of Toronto, Toronto, ON M5S 1A1, Canada3Department of Laboratory Medicine and Pathobiology, University of Toronto, Toronto, ON M5S 1A1, Canada4Odette Cancer Centre, Sunnybrook Health Sciences Centre, Toronto, ON M4N 3M5, Canada*Correspondence: katarzyna.jerzak@sunnybrook.ca

 

## 1. Background 

Brain metastases (BrM) are a major cause of morbidity and mortality in women with breast cancer. Immunotherapy has the potential for intracranial efficacy among patients with breast cancer BrM since intracranial response to immunotherapy has been observed in other solid tumors. The aim of the study is to analyze the immunohistochemical expression of programmed death ligand 1 (PD-L1), a predictive biomarker of response to immunotherapy, in breast cancer BrMs.

## 2. Methods 

A retrospective cohort study of consecutive patients with metastatic breast cancer BrM who underwent surgery for BrM at Sunnybrook Health Sciences Centre between July 1999 and June 2013 were identified through the Anatomic Pathology departmental database. A tissue microarray using 1um cores was obtained. PD-L1 expression by immunohistochemistry (IHC) was assessed on BrM samples in triplicate; PD-L1 positive status was defined as PD-L1 expression ≥1% on tumor infiltrating cells as a percentage of tumor area using Ventana SP142 antibody. Estrogen receptor (ER), progesterone receptor (PR) and human epidermal growth factor receptor (HER2) status was determined using 2018 ASCO/CAP guidelines. 

## 3. Results

The median patient age at the time of BrM diagnosis was 53 (range 32–85). In our overall cohort, PD-L1 expression was identified in nine out 61 (14.7%) breast cancer BrM. ER, PR and HER2 status was available for BrM in 60 out of 61 patients. Patients with triple negative breast cancer were most likely (*n*= 3/12, 25%) and those with HER2+ breast cancer were least likely (*n* = 3/28, 10.7%) to have PD-L1 positive BrM. Among patients with hormone receptor positive/HER2- breast cancer, 15% (*n* = 3/20) of BrM were PD-L1 positive. 

## 4. Conclusions 

One in seven patients in our cohort had PD-L1 positive BrM; this proportion was highest (25%) among those with triple negative disease. Hence, there is rationale to include patients with breast cancer BrM in clinical trials evaluating efficacy of immunotherapy. 

## 

*15_CAMO_2021 (Award Recipient)*

**Outcomes of Elderly Patients with Unresectable Stage 3 Nsclc Treated with Definitive Chemoradiation with or without Durvalumab**

## 

RyanMalcolmWeissJessicaFusco FaresAlineTsaoMing SoundLiuGeoffreyBradburyPenelope A.LeighlNatasha B.ShepherdFrances A.SacherAdrian G.LauSally C.M.*Princess Margaret Cancer Centre, University Health Network, University of Toronto, Toronto, ON M5G 2M9, Canada*Correspondence: Sally.Lau@uhn.ca

 

## 1. Introduction

The recent addition of durvalumab after chemoradiation (CRT) in unresectable stage 3 non-small cell lung cancer (NSCLC) significantly improves survival. The benefit of CRT in elderly patients is controversial given its increased toxicity. However, patients cannot receive durvalumab unless CRT was given. We sought to investigate the outcomes of elderly patients treated with CRT. 

## 2. Methods

We conducted a review of all stage 3 NSCLC patients treated with CRT between 2018 and 2020. Patients were analyzed based on age: <70 years, ≥70 years. Endpoints evaluated were treatment patterns, toxicity, progression free survival (PFS) and overall survival (OS). 

## 3. Results

We identified a total of 106 patients: 40 patients ≥70 years (70–89) and 66 patients <70 years (34–69). Patients were fit: ECOG 0–1 (98%/99%), mean Charlson comorbidity index (CCI) (1.3/1.1) in elderly vs. young patients; *p* > 0.05. All other baseline characteristics including PD-L1 expression were similar. The chemotherapy regimens and dose intensity were similar. However, patients ≥70 were less likely to receive all planned number of cycles (*p* = 0.05). There were two treatment related deaths from CRT in young and none in the elderly patients. At the completion of CRT, 72% of elderly and 70% of young patients received durvalumab. The incidence of grade ≥3 immune-related adverse events was 8% in elderly patients and 5% young patients; *p* = 0.68. Median PFS was similar between elderly and young patients (17.6 vs. 10.2 months respectively; *p* = 0.08), even after adjusting for the CCI (HR 0.60; *p* = 0.08). The 12-month OS rates are also similar (*p* = 0.93): 86% in elderly and 83% in young patients. 

## 4. Conclusions

Definitive CRT followed by durvalumab is tolerable in elderly patients ≥70 years with a non-significant trend towards better PFS in elderly patients. All patients should undergo comprehensive oncologic assessment to determine if curative intent treatment can be delivered to avoid undertreatment. 

## 

*16_CAMO_2021*

**Impact of Smoking on Response to Immunotherapy in *KRAS* Mutant Non-Small Cell Lung Cancer**

## 

SharmaKieran*KuangShelleyTsaoMing SoundShepherdFrances A.BradburyPenelope A.LiuGeoffreyLeighlNatasha B.SacherAdrian G.LauSally C.M.Princess Margaret Cancer Centre, University Health Network, University of Toronto, Toronto, ON M5G 2M9, Canada*Correspondence: kr.sharma@mail.utoronto.ca

 

## 1. Background

Immune checkpoint inhibitors (ICI) are highly effective in the management of advanced non-small cell lung cancer (NSCLC). Non-smokers appear to derive less benefit from ICI, often attributed to the higher likelihood of a primary driver mutation. However, the variation in responses to ICI cannot be explained by oncogene addiction alone. We sought to investigate the impact of smoking among *KRAS* driven NSCLC.

## 2. Methods

We conducted a review of patients with *KRAS* mutant advanced NSCLC who have received at least one cycle of ICI. Primary outcomes were overall response rates (ORR) and progression free survival (PFS).

## 3. Results

We identified 91 patients with *KRAS* mutant NSCLC who were treated with ICI: 23 never/53 former/15 current smokers with similar distributions of age, ethnicity, and tumor histology. There was trend towards a higher proportion of females among never smokers (*p* = 0.09). Smoking history also was associated with a trend (*p* = 0.06) to high PD-L1 expression (≥50%): 27%/53%/62% and significantly higher rates of TP53 co-mutations: 36%/46%/80% (*p* = 0.03) among never/former/current smokers, respectively. Transversion mutations account for 76%/81%/93% of never/former/current smokers; *p* = 0.13. ORR were higher among smokers: 13%/34%/80% among never/former/current smokers (*p* = 0.001) and remained significant even after adjusting for PD-L1 expression. Median PFS was associated with smoking on univariate analysis (2.9 vs. 4.9 vs. 26.8 months in never/former/current smokers; *p* = 0.02), but the association was lost after adjusting for PD-L1 expression. 

## 4. Conclusions

Never smokers with *KRAS* mutant NSCLC appear to derive less benefit from ICI. The differences in PD-L1 expression and rates of TP53 co-mutations, which are surrogate markers of response, suggest that the underlying tumor immune microenvironment (TME) among smokers and non-smokers, even in the presence of the same oncogene, is fundamentally different. Correlative efforts using serial plasma samples are ongoing to help understand the impact of smoking on the TME and response to ICI.

## 

*17_CAMO_2021*

**Ogivri^®^ versus Herceptin^®^—“Real World” pCR Rates in Patients with HER2+ Breast Cancer Treated with Neoadjuvant Chemotherapy Plus Trastuzumab from Alberta, Canada**

## 

YangCharlie^1^*†KhwajaRaida M.^2^†KingKaren^2^NixonNancy A.^1^TangPatricia A.^1^LupichukSasha^1^1Department of Medical Oncology, University of Calgary, Calgary, AB T2N 1N4, Canada2Department of Medical Oncology, University of Alberta, Edmonton, AB T6G 2R3, Canada*Correspondence: charlie.yang@albertahealthservices.ca†Co-first authors.

 

## 1. Objective

This retrospective study compared the pathological complete response (pCR) rates of trastuzumab-dkst (Ogivri^®^) to Herceptin^®^ in the neoadjuvant setting for HER2+ early breast cancer (EBC) in Alberta.

## 2. Methods

Neoadjuvant patients with HER2+ EBC treated with Herceptin^®^ from November 2018–October 2019 and Ogivri^®^ from December 2019–September 2020 were identified. There was no crossover between products. Logistic regression was used to control for variables potentially associated with pCR: trastuzumab product (Ogivri^®^ vs. Herceptin^®^), age (<40 vs. 40+), pre-operative T (T1/2 vs. T3/4) and N stage (negative vs. positive), grade (I/II vs. III), HR status (ER and/or PR positive vs. ER/PR negative), HER2 (3+ vs. SISH+), chemotherapy (anthracycline containing vs. not), and chemotherapy completion (yes vs. no).

## 3. Results

136 patients were identified (56% Herceptin^®^; 43% Ogivri^®^) and there were no significant differences in baseline characteristics except more patients in the Ogivri^®^ group were clinically N negative; 39% vs. 14.3% Herceptin^®^ (*p* = 0.001). pCR was 35.6% for patients treated with Ogivri^®^ versus 40.3% with Herceptin^®^ (*p* = 0.598). In the logistic regression model, there was no significant difference in the odds of a pCR for patients treated with Ogivri^®^ versus Herceptin^®^ after controlling for the variables selected a priori (OR 1.1, 95% CI 0.5–2.4, *p* = 0.850). There was a trend for decreased odds of pCR for anthracycline use (OR 0.72, 95% CI 0.3–1.6, *p* = 0.417).

## 4. Conclusions

pCR rates were similar for patients treated with Ogivri^®^ compared to Herceptin^®^ in our real-world study of HER2+ neoadjuvant EBC and comparable to pivotal phase 3 trials. For a 65 kg patient, the estimated cost savings of Ogivri^®^ therapy is $22,000, and approximately $240–300 for a non-anthracycline chemotherapy backbone.

## 

*18_CAMO_2021*

**The Effect of the COVID-19 Pandemic on the Evolution of Cancer Care in Nova Scotia**

## 

SheridanMargaret*ColwellBruceDruckerArikMacfarlaneRobynRaysonDanielSnowStephanieWoodLoriRamjeesinghRaviDivision of Medical Oncology, Dalhousie University, Halifax, NS B3H 2Y9, Canada*Correspondence: margaret.sheridan@dal.ca

 

## 1. Objective

To review Medical Oncology (MO) workload in a local context and evaluate the impact of COVID-19.

## 2. Methods

All patient encounters (new patient consults, follow-up visits (F/U), telephone toxicity assessments, and chart checks) were identified over a 3-month period (February through April) across a 6-year interval (2014–2019) and extrapolated to derive an estimate of annual workload. This data was then analyzed based on type of encounter and disease site. The same data was collected over one month from mid-March to mid-April 2020, during the province-wide COVID-19 lockdown measures. 

## 3. Results

In 2014, there were an estimated 2052 new consults, which increased to 2484 by 2019 (21.1% increase). The number of F/U increased from 9312 to 10,532 (13.1%). Virtual care (VC), which includes chart reviews and virtual consults, and telephone toxicities increased by 24.6% and 238.1% respectively over a similar time span.

VC accounted for 41.2% of the care provided in 2018 and 45.2% of the care provided in 2019, which increased to 79.9% in 2020, during a sampled time period during the COVID lockdown. A proportional change was observed amongst different treatment sites. 

VC provided by immunotherapy treaters increased from 49.9% in 2018 to 53.8% in 2019 and to 85.3% in 2020. A larger increase was seen in the non-immunotherapy treaters, who provided only 34.1% of VC in 2018, 34.8% in 2019, but 73.5% in 2020, more than doubled what was observed in the previous two years. 

## 4. Conclusions

MO workload has increased over time, with more new consults, increasing time spent in follow up and delivery of VC, due to the changing landscape of cancer care and now more poignantly, in the wake of the COVID-19 pandemic. This metric requires recognition in effort to ensure delivery of optimal patient care moving forward. 

## 

*19_CAMO_2021*

**Population-Based Impacts of New Therapies on Outcomes for Stage IV Non-Small Cell Lung Cancer**

## 

RittbergRebekah^1^,^2^*BucherOliver^3^XueLin^3^AurangzebZeb^3^BanerjiShantanu^1^,^2^,^4^DaweDavid E.^1^,^2^,^4^1Department of Internal Medicine, University of Manitoba, Winnipeg, MB R3T 2N2, Canada2CancerCare Manitoba, Department of Hematology and Medical Oncology, Winnipeg, MB R3E 0V9, Canada3CancerCare Manitoba, Department of Epidemiology, Winnipeg, MB R3E 0V9, Canada4Research Institute in Oncology and Hematology at CancerCare Manitoba, Winnipeg, MB R3E 0V9, Canada*Correspondence: rrittberg@cancercare.mb.ca

 

## 1. Objective

Evaluate real world, population-based treatment patterns and outcomes of Stage IV non-small cell lung cancer (NSCLC) to assess changes in treatment patterns and survival.

## 2. Methods

A retrospective cohort analysis was completed to evaluate de novo Stage IV NSCLC diagnosed in Manitoba from 2006 to 2015. We evaluated treatment received (not seen by specialist, saw a specialist but did not receive therapy, radiation therapy (RT) only, and systemic therapy (mutation unknown and known)) and treatment era of diagnosis (2006–2009, 2010–2013 and 2014–2015). Multivariable logistic regression assessed systemic therapy predictors. Kaplan-Meier curve and Cox proportional hazard models evaluated overall survival (OS). 

## 3. Results

3601 patients were diagnosed with Stage IV NSCLC, 53% male. Only 21% received systemic therapy, mean age of 62. Within the cohort, 973 (27%) patients did not see a specialist, 610 (17%) saw a specialist but did not receive therapy, 1248 (35%) only received RT, and 771 (21%) received systemic therapy (17% mutation status unknown and 4% known). Younger patients and those with confirmed histology were more likely to see a specialist and receive treatment, each (*p* < 0.001). Patients who received systemic therapy had lower comorbidity and higher income quintile, each (*p* < 0.001). Median OS did not differ between treatment era with median OS of 3.0, 2.9 and 2.8 months for 2006–2009, 2010–2013 and 2014–2015 respectively, *p* = 0.082. When survival analysis was restricted to patients who received systemic therapy, median OS improved by era to 10.9, 11.2 and 15.6 months respectively, *p* = 0.001. Variables found to be independently associated with survival included treatment type, age, sex and comorbidity. 

## 4. Conclusions

Improved systemic therapy and molecular testing has improved OS for patients who receive systemic therapy. However, due to the large proportion of Stage IV NSCLC patients who never receive systemic therapy we do not see improved survival at a population level between treatment eras. 

## 

*20_CAMO_2021*

**Increased Survival in Patients with Melanoma Who Develop Immune Related Adverse Events: A Real-World Retrospective Study**

## 

HolsteadRyan^1^*KartoloAdi^1^HopmanWilma^2^BaetzTara^1^1Department of Oncology, Queen’s University, Kingston, ON K7L 3N6, Canada2Department of Medicine, Queen’s University, Kingston, ON K7L 3N6, Canada*Correspondence: ryangordon.holstead@kingstonhsc.ca

 

## 

Some clinical trials have described improved outcomes in patients who develop immune-related adverse events (irAEs), while receiving immune checkpoint inhibitors for advanced melanoma. It is unknown if this effect would be seen in a real world population.

This is a single-center retrospective analysis of all patients receiving single agent PD-1 inhibitor for unresectable stage III or stage IV melanoma between 2012 and 2018. The majority of patients had cutaneous melanoma and were elderly (put in median and range). 33.3% were BRAF mutated and 22% of patients had brain metastases at presentation. Of the 87 patients included in this analysis, 48 (55%) developed at least one irAE. Dermatologic toxicities were the most common irAE. The median time to develop any irAE was 12 weeks. Only one patient died of immune related toxicity.

Overall survival in the population of patients that had an irAE was significantly greater than those that did not have any toxicity (21.1 vs. 7.5 months; *p* < 0.001). The development of endocrine toxicity had the strongest correlation with survival.

A high grade of toxicity (NCI CTC V.5) did not correlate with survival outcome and the greatest correlation was seen in patients with grade I toxicity. The development of multiple toxicities did not correlate with survival. In patients with multiple toxicities the type of irAE that presented initially did not impact the outcome. These findings add to the growing body of literature suggesting an association between immune related toxicity and immune-checkpoint inhibitor efficacy, while suggesting that this benefit may depend on type of toxicity and severity. 

## 

*21_CAMO_2021*

**First-Line Treatment with a Cyclin-Dependent Kinase 4/6 Inhibitor Combined with an Aromatase Inhibitor for Hormone Receptor Positive, Human Epidermal Growth Factor Receptor-2 Negative Metastatic Breast Cancer: Population-Based Outcomes for Patients Treated in Alberta, Canada**

## 

AmaroCarla P.^1^*BatraAtul^2^LupichukSasha^1^1Tom Baker Cancer Centre, University of Calgary, Calgary, AB T2N 1N4, Canada2All India Institute of Medical Sciences, New Delhi 110029, India*Correspondence: amarocal@hotmail.com

 

## 1. Objective

To describe population-based outcomes for first-line treatment with a cyclin-dependent kinase 4/6 inhibitor (CDK4/6i) combined with an aromatase inhibitor (AI) in patients with hormone receptor (HR)-positive, human epidermal growth factor receptor-2 (HER2)-negative metastatic breast cancer (MBC) patients in Alberta.

## 2. Methods

All patients who were prescribed CDK4/6i + AI from January 2016 through June 2019 were included. Descriptive statistics were used to summarize patient demographics, tumor and treatment characteristics. Survival distributions were estimated using the Kaplan-Meier method. Multivariate analysis (MVA) using a Cox proportional hazards model was constructed to examine associations between potentially prognostic clinical variables and progression free survival (PFS). 

## 3. Results

A total of 316 patients were included. Median age was 61 years, 82% were postmenopausal women, 39% had de novo MBC, and 48% had non-visceral disease. Palbociclib was prescribed in 94% of patients. The CDK4/6i was dose-reduced upfront or during treatment in 47%. While 70% of the patients discontinued treatment due to progression, 30% stopped due to toxicity/patient preference/physician recommendation. With a median follow-up of 28.1 months, the median PFS was 37.9 months (95% CI, 26.7-NR). In the MVA, PR-negative tumour (HR, 2.37; 95% CI, 1.45–3.88; *p* = 0.001) and dose reduction of the CDK4/6i (HR, 1.51; 95% CI, 1.06–2.16; *p* = 0.022) predicted worse PFS. Median overall survival (OS) was not reached. The 30-month and 36-month OS rates were 74% and 68%, respectively. Of patients who progressed, 89% received second-line treatment (chemotherapy in 46%, single agent hormonal therapy in 35%, hormonal therapy plus a targeted agent in 15%, and other in 4%). Median time on second line chemotherapy was 9.0 (5.8–17.6) months and second line hormonal therapy ± targeted agent was 4.0 (3.4–8.6) months (*p* = 0.012).

## 4. Conclusions

CDK4/6i + AI as first-line treatment for HR-positive, HER2-negative MBC in Alberta is justified based on favorable PFS and early OS outcomes.

## 

*22_CAMO_2021*

**A Systemic Review and Meta-Analysis of Combination Chemo-Immunotherapy in the First Line Treatment of Extensive Stage Small Cell Lung Cancer (ES-SCLC)**

## 

FebbraroMichela*SathiyapalanAraniPondGregoryEllisPeter M.Juravinski Cancer Center, Hamilton, ON L8V 5C2, Canada*Correspondence: michela.febbraro@medportal.ca

 

## 1. Background

The standard of care treatment for ES-SCLC with platinum-etoposide chemotherapy has not changed in 30 years. Recently, several studies have demonstrated improved progression-free survival (PFS) and overall survival (OS) with the use of combined chemo-immunotherapy in the first line treatment of ES-SCLC. A systematic review and meta-analysis assessing the magnitude of these improvements was conducted. 

## 2. Methods

MEDLINE, EMBASE and the Cochrane library were searched between 1 January 2010 and 10 June 2020 and ASCO, ESMO, and WCLC conference proceedings from 2018 to 2020 were searched. Randomized controlled trials comparing chemo-immunotherapy to platinum-etoposide or platinum-paclitaxel chemotherapy alone in untreated ES-SCLC were included. Trials evaluating treatment beyond the first line setting were excluded. Outcomes of interest included PFS, OS, objective response rate (ORR), duration of response (DoR), toxicity and health-related quality of life (HRQoL). 

## 3. Results

2705 studies and abstracts were initially identified, with six studies (33 publications) included in the final analysis. PFS (HR 0.81, 95% CI 0.75–0.87) and OS (HR 0.82, 95% CI 0.76–0.89) were significantly improved for patients randomized to chemo-immunotherapy compared with chemotherapy. Between-group heterogeneity being low (I^2^ 0%) for both outcomes. Pre-specified subgroup analysis demonstrated no evidence of any differential effect in outcomes between PD-1/PD-L1 inhibitors and CTLA-4 inhibitors. There was no difference in ORR (risk ratio 1.04, 95% CI 0.98–1.10) or DoR (mean difference 0.09 months, 95% CI −0.13 to 0.32). All grade adverse events (pooled risk ratio 1.03, 95% CI 1.01–1.06) or grade 3–4 adverse events (risk ratio 1.06, 95% CI 0.99–1.13) were similar between groups. 

## 4. Conclusions

The addition of immunotherapy to chemotherapy in the first line treatment of ES SCLC results in a 19% risk reduction in disease progression and 18% risk reduction in death. Survival improvements were associated with a minimal increase in toxicity and may represent an improvement beyond standard of care. 

## 

*23_CAMO_2021*

**Impact of Age, Comorbidity and Polypharmacy on Treatment and Survival for Aggressive Non-Hodgkin Lymphoma**

## 

TapleyLaura^1^*SkrabekPamela^1^LambertPascal^2^BravoJenniebie^2^DeckerKathleen^3^CzaykowskiPiotr^1^TurnerDonna^2^St. JohnPhil^1^DaweDavid E.^1^,^3^1Department of Internal Medicine, University of Manitoba, Winnipeg, MB R3A 1R9, Canada2Epidemiology and Cancer Registry, Research Institute in Oncology and Hematology, CancerCare Manitoba, Winnipeg, MB R3E 0V9, Canada3Research Institute in Oncology and Hematology, CancerCare Manitoba, Winnipeg, MB R3E 0V9, Canada*Correspondence: umtaplel@myumanitoba.ca

 

## 1. Objective

To determine if age, medication count, and co-morbidity independently impact receipt of chemotherapy and survival in aggressive Non-Hodgkin’s Lymphoma (NHL).

## 2. Methods

We identified patients diagnosed with aggressive NHL aged >18 years from 2004–2015 in the Manitoba Cancer Registry using morphology codes. Demographics, stage, NHL type, comorbidities, polypharmacy, and chemotherapy were obtained from population-based provincial databases. Overall survival (OS) was calculated using Kaplan–Meier curves. Cox proportional hazards regression models were constructed to determine interaction of age with other variables. Multi-variable logistic regression was used to examine receipt of chemotherapy and interaction with age.

## 3. Results

The population-based cohort of 1073 patients with aggressive NHL was stratified by age (<50 [*n* = 86], 50–59 [153], 60–69 [258], 70–79 [295], 80+ [281]) with 704 treated with systemic chemotherapy. Treatment rates decreased with age and medication count. Median OS decreased with age among treated patients and was 6.09 years (95% CI 4.54–7.45) in treated and 0.18 years (95% CI 0.13–0.24) in untreated patients. OS for treated patients <50 was not reached and decreased with age. OS in untreated patients was uniformly poor. Multivariate analyses showed individuals with increasing age, stage III, unknown stage, non-DLBCL histology, and polypharmacy were less likely to receive chemotherapy. For receipt of chemotherapy, there were no interactions of age with other variables. In treated patients, age and stage were associated with poorer survival. No interactions between age and other variables substantially impacted on survival.

## 4. Conclusions

OS in aggressive NHL diminishes with age, but is longer in those receiving chemotherapy. Comorbidity and polypharmacy influenced receipt of chemotherapy and OS. Polypharmacy was associated with lower likelihood of treatment, while comorbidity was not a predictor of either treatment or OS. Comorbidity and medication count did not statistically interact with age.

## 

*24_CAMO_2021*

**Integrating Machine Learning Models into the Management of Vasomotor Symptoms in Breast Cancer Patients**

## 

ColeKatherine Marie^1^*ClemonsMark^1^El EmamKhaled^2^,^3^LarocqueGail^4^MacDonaldFiona^4^VandermeerLisa^5^HuttonBrian^6^PiperArdelle^7^PondGreg^8^McGeeSharon^1^1Department of Medicine, Division of Medical Oncology, University of Ottawa, Ottawa, ON K1N 6N5, Canada2School of Electrical Engineering and Computer Science, University of Ottawa, Ottawa, ON K1N 6N5, Canada3Faculty of Medicine, Children’s Hospital of Eastern Ontario, Ottawa, ON K1H 8L1, Canada4The Ottawa Hospital Cancer Centre, Ottawa, ON K1H 8L6, Canada5Cancer Therapeutics Program, Ottawa Hospital Research Institute, Ottawa, ON K1H 8L6, Canada6Clinical Epidemiology Program, The Ottawa Hospital Research Institute, Ottawa, ON K1H 8L6, Canada7University of Ottawa Health Services, Ottawa, ON K1N 6N5, Canada8Department of Oncology, McMaster University, Hamilton, ON L8S 4L8, Canada*Correspondence: kcole077@uottawa.ca

 

## 1. Objective

Vasomotor symptoms (VMS) including hot flashes and night sweats are common in early breast cancer (EBC) and can lead to reduced quality of life and treatment discontinuation. We aimed to determine how EBC patients defined effective control of VMS. We also used patient survey data to develop a machine learning model predicting the risk of developing distressing VMS.

## 2. Methods

We conducted a patient survey in women who experienced hot flashes during treatment for EBC at The Ottawa Hospital. For each participant, we collected 28 variables relating to demographics, menopausal status, and previous treatments for both EBC and VMS. We quantified the frequency, intensity, and temporality of VMS. Distress from VMS was quantified on a ten point scale using the validated Hot Flush Rating Scale. A gradient boosted tree machine learning model was trained to predict the distress scale based on the variables collected. 

## 3. Results

Between June 4, 2020 and January 8, 2021, 301 patients, average age of 56 years (range 23–83), responded to the survey. The mean number of hot flashes per day was 5 (range 0–80), and 47% of respondents rated their level of distress from VMS as moderate to severe. The most bothersome symptoms reported were sweats and disturbed sleep. Most respondents (157/288, 55%) indicated that they would consider a treatment effective if it controlled nocturnal symptoms. The model trained to predict distress level reached an 85% accuracy on a 10-point classification task. 

## 4. Conclusions

Appropriately managing nocturnal symptoms is the primary concern of EBC patients experiencing VMS. We demonstrated that a machine learning model can accurately predict the amount of distress experienced by EBC patients with VMS. Machine learning has future applications in the prediction and assessment of toxicities from cancer therapies.

## 

*25_CAMO_2021*

**The Impact of the Modified Frailty Index on Clinical Outcomes for Stage IV Non-Small Cell Lung Cancer Patients (NSCLC) Receiving Chemotherapy**

## 

MathurShivani^1^*PrinceLaura^1^BucherOliver^2^XueLin^2^BanerjiShantanu^3^,^4^,^5^DaweDavid E.^3^,^4^,^5^1Max Rady College of Medicine, University of Manitoba, Winnipeg, MB R3T 2N2, Canada2Department of Epidemiology, CancerCare Manitoba, Winnipeg, MB R3E 0V9, Canada3Department of Internal Medicine, University of Manitoba, Winnipeg, MB R3T 2N2, Canada4Department of Medical Oncology and Hematology, CancerCare Manitoba, Winnipeg, MB R3E 0V9, Canada5Research Institute in Oncology and Hematology, CancerCare Manitoba, University of Manitoba, Winnipeg, MB R3T 2N2, Canada*Correspondence: mathurs3@myumanitoba.ca

 

## 1. Objective

To evaluate any association between the Modified Frailty Index (mFI) and clinical outcomes for metastatic NSCLC patients receiving cytotoxic chemotherapy.

## 2. Methods

We conducted a retrospective cohort study of all Stage IV NSCLC patients diagnosed in Manitoba between 1 January 2011 and 31 December 2016 who then received first-line cytotoxic chemotherapy. We reviewed CancerCare Manitoba charts to assign patients a mFI score based on comorbidities and to collect data on toxicity, cancer response, and progression date. Descriptive statistics were used to characterize the cohort and evaluate toxicity. Kaplan-Meier methods were used to evaluate progression-free survival (PFS) and overall survival (OS), followed by multivariable Cox proportional hazards models.

## 3. Results

In our cohort of 426 (mFI 0/1–2/3+ = 175/196/55) patients, there was no significant association between a higher mFI score and the increased incidence of chemotherapy-related toxicities. Patients with mFI 0 experienced more frequent thromboses (*p* = 0.022) and a trend towards less nausea or vomiting (*p* = 0.059). PFS by mFI category was 4.9 months (mFI = 0), 5.39 months (mFI = 1–2), and 5.95 months (mFI = 3+). Median OS between groups was 9.0 months (mFI = 0), 8.6 months (mFI = 1–2), and 10.8 months (mFI = 3+). There was no significant difference in PFS and OS among frailty groups, with *p* values of 0.98 and 0.21 respectively. Poorer ECOG scores, number of metastatic sites, and the absence of a driver mutation were independently associated with poorer PFS and OS. Male sex and not completing chemotherapy were associated with worse OS.

## 4. Conclusions

This study is the first to investigate the use of the mFI as a frailty tool in metastatic NSCLC patients receiving cytotoxic chemotherapy. The mFI does not appear to be associated with treatment-related toxicities, PFS, or OS in patients with metastatic NSCLC receiving first-line cytotoxic chemotherapy. 

## 

*26_CAMO_2021*

**Real World Use of Lanreotide in Management of Neuroendocrine Tumors**

## 

SiddiquiZeba^1^*MargineanHoria^2^,^3^AsmisTimothy^2^,^3^VickersMichael^2^,^3^GoodwinRachel^2^,^3^1Internal Medicine Residency Program, The Ottawa Hospital (TOH), Ottawa, ON K1H 8L6, Canada2The Ottawa Hospital Cancer Centre (TOHCC), Ottawa, ON K1H 8L6, Canada3Ottawa Hospital Research Institute (OHRI), Ottawa, ON K1H 8L6, Canada*Correspondence: zsiddiqui@toh.ca

 

## 1. Objective

A majority of neuroendocrine tumors (NETs) arise from the gastrointestinal tract and present with metastases. Treatment is often with somatostatin analogues (SSA) such as lanreotide in the first line setting. There is a paucity of Canadian data on use of lanreotide. We aimed to study real world use of lanreotide in management of NETs. 

## 2. Methods

We performed a single-site retrospective chart review of all patients (*n* = 69) on lanreotide for NETs at The Ottawa Hospital Cancer Center. We studied patient characteristics and provider practices surrounding lanreotide use. 

## 3. Results

68% of patients were male, median age was 64 years (range 35–93 years) and 57% of patients had confirmed grade 1 or 2 primary. Majority of patients had either gastrointestinal (48%) or pancreatic (32%) NET. 62% of patients presented in metastatic setting and the liver (50%) was the most common site. 96% of patients were started on standard dose, 120 mg q28d, and 88% were maintained on this dose. In 83% of cases lanreotide was first line of treatment. In 10% of patients lanreotide was stopped due to low grade intolerance (abdominal pain, hyperglycemia, diarrhea). Intention of treatment was tumor control, with or without symptom control, in 96% of patients. Only 4% of patients were offered lanreotide for symptom control alone. 

## 4. Conclusions

In keeping with Canadian guidelines, SSA therapy was often used in the first line setting. When lanreotide is used, the majority of patients are initiated and continued on the standard dose with the goal of tumour control. In the future, it will need to be studied if dose escalation is used more often given the recent Clarinet Forte data showing that increasing the dose frequency of lanreotide from monthly to bi-monthly resulted in progression-free survival of 8.3 and 5.6 months in progressive midgut NETs and pancreatic NETs, respectively.

## 

*27_CAMO_2021*

**Recurrent Disease in Patients with Stage III Melanoma in the Era of Adjuvant Immune and Targeted Therapy**

## 

LoVictor C. K.^1^FrancescuttiValerie A.^2^,^3^McWhirterElaine^3^,^4^LeeLinda M.^4^,^5^*1Department of Medicine, McMaster University, Hamilton, ON L8S 4L8, Canada2Department of Surgery, McMaster University, Hamilton, ON L8S 4L8, Canada3Juravinski Cancer Centre, Hamilton Health Sciences, Hamilton, ON L8V 5C2, Canada4Department of Oncology, McMaster University, Hamilton, ON L8S 4L8, Canada5Walker Family Cancer Centre, Niagara Health, St. Catharines, ON L2S 0A9, Canada*Correspondence: linda.lee@niagarahealth.on.ca

 

## 1. Objective

To describe characteristics of stage III melanoma patients with recurrent disease following surgical management and evaluate the impact of adjuvant treatment on recurrence.

## 2. Methods

A multicenter retrospective chart review of patients with pathologically confirmed Stage III cutaneous melanoma seen at either the Juravinski Cancer Centre or Walker Family Cancer Centre from 1 January 2017 to 31 December 2019.

## 3. Results

There were 137 patients with Stage III melanoma: 18% IIIA, 22% IIIB, 52% IIIC, and 8% Stage IIID as per the 8th American Joint Committee on Cancer (AJCC) 2018 staging system. 102 (74%) patients had sentinel lymph node biopsy as part of initial surgical therapy, and 31 (23%) had radical nodal dissection. Sixty seven (49%) patients received adjuvant therapy, of which 49 (73%) had immunotherapy, 17 (25%) received BRAF-targeted therapy, and two (3%) had interferon. 54 (39%) patients developed recurrent disease, with a median time to recurrence of 8.7 months (IQR: 1.4–14.9). By pathologic subgroup, recurrence rates were 12%, 37%, 48%, and 55% for Stage IIIA, IIIB, IIIC, and IIID respectively. There were 31 (57%) loco-regional recurrences and 23 (43%) distant recurrences. The recurrent rates were 63% in patients who did not have adjuvant treatment and 37% in those who had adjuvant therapy, with a median time-to-recurrence of 8.2 and 9.0 months respectively. There were 13 patients who recurred on adjuvant treatment and seven patients who recurred following completion of adjuvant treatment. First recurrences were detected by patients, clinicians, computed tomography and nodal ultrasound surveillance in 43%, 20%, 28% and 9% of cases, respectively.

## 4. Conclusions

Recurrences in Stage III melanoma occur early, often within a year, with higher rates of loco-regional rather than distant disease. Recurrence rates were lower in those who received adjuvant therapy. The majority of recurrences were patient-detected, highlighting the importance of patient education regarding self-monitoring.

## 

*28_CAMO_2021*

**Workload Assessment among Medical Oncologists in Atlantic Canada**

## 

FaourElizabeth^1^*ChampionPhilip^2^ColwellBruce^3^GraySamantha^4^HarbMohammed^5^McCarthyJoy^1^St-HilaireEve^6^RamjeesinghRavi^3^1Discipline of Oncology, Memorial University, St. John’s, NL A1B 3V6, Canada2Division of Medical Oncology, Dalhousie University, Charlottetown, PE C1A 8T5, Canada3Division of Medical Oncology, Dalhousie University, Halifax, NS B3H 4R2, Canada4Division of Medical Oncology, Dalhousie University, Saint John, NB E2L 4L5, Canada5Division of Medical Oncology, Dalhousie University, Moncton, NB E1C 6Z8, Canada6Centre Hospitalier Universitaire, Dr-Georges-L-Dumont, Moncton, NB E1C 2Z3, Canada*Correspondence: elizabethfaour@gmail.com

 

## 1. Objective

Globally, workload among medical oncologists has been identified as an issue requiring attention. Atlantic Canadian data has not previously been gathered, thus we set out to examine workload and human resources among Atlantic Canadian Medical Oncologists (MO’s).

## 2. Methods

To assess workload and resource allocations in Atlantic Canada, a questionnaire was developed and sent to representatives from each cancer centre in Atlantic Canada including St. John’s NL; Halifax NS; Saint John NB; Moncton NB (Horizon Health and Vitalité); and Charlottetown PEI. Data was compiled and workload deficits calculated using the 2000 Canadian Task Force recommendations as targets. 

## 3. Results

At the time of data collection, there were 46 MO’s in Atlantic Canada. All sites have workload levels above the recommended 160 new consults per year per MO, with average workload varying from 165 to 250. MO deficits calculated for each site varied from 0.1 to 3.2 full time equivalent (FTE). There are also gaps in nursing and pharmacy resources; 2/6 sites do not have a primary nursing model, and 2/6 sites do not have pharmacists providing supports in clinic.

## 4. Conclusions

Atlantic Canada is no exception to the global issue of workload burden among MO’s. Given the changing landscape of medical oncology wherein patients live longer with metastatic disease, the task force recommendations from 2000 probably no longer represent reasonable targets, meaning the deficits are larger than we know. Given the aging population, and the increasing rates of cancer diagnoses as well as therapeutic options, we can expect workforce gaps to widen with time. Additionally, in the context of a culture where physician burnout is increasingly prevalent, excess workload is especially problematic. Workload among Medical Oncologists is an issue that needs to be addressed to avoid the crisis in systemic therapy that the task force initially set out to prevent in 2000.

## 

*29_CAMO_2021*

**Real World Outcomes of First Line Pembrolizumab Monotherapy in Advanced Non-Small Cell Lung Cancer Patients**

## 

ChuRyan W.^1^*VegasAntonio^2^LeighlNatasha^3^GorryClaire^4^PowerDerek^5^1School of Medicine, University College Cork, T12 YN60 Cork, Ireland2Business Administration, University Carlos III de Madrid, 28903 Madrid, Spain3Medical Oncology, Princess Margaret Cancer Centre, Toronto, ON M5G 1Z5, Canada4Pharmacology and Therapeutics, Trinity College, Dublin 2 DO2 PN40, Ireland5Medical Oncology, Cork University Hospital, T12 YE02 Cork, Ireland*Correspondence: 118104301@umail.ucc.ie

 

## 1. Objective

To explore the effectiveness of first line pembrolizumab monotherapy for advanced non-small cell lung cancer (NSCLC) in real world patients (RWP) from a regional oncology setting in Ireland. 

## 2. Methods

This was a single-centre, retrospective, observational study. Patients (pts) treated at Cork University Hospital (CUH) with first line pembrolizumab monotherapy for advanced (stage IIIB/IV) NSCLC between the years 2017–2019 were eligible. Pts were identified using pharmacy dispensing records. Pt demographics, progression free survival (PFS), overall survival (OS) and adverse events were collected from pt records. These data were then compared with pts that received pembrolizumab monotherapy as part of the Phase III Keynote-024 randomised clinical trial (RCT). 

## 3. Results

In total, 85 pts received pembrolizumab at CUH between 2017–2019. 25 pts were deemed eligible for this study. Reasons for exclusion include: ineligible diagnosis (52 pts), received pembrolizumab as second line therapy (7 pts), and received pembrolizumab as combination therapy (1 pt). Median follow up was 10.5 months. PFS at 6 months from the RWP and RCT pts were 56% and 61% respectively, while OS was 76% and 80% respectively. 12% (3 pts) of RWP discontinued treatment due to adverse events while this occurred in 7.1% of RCT pts. Key differences between the two cohorts included ECOG performance status (ECOG ≥ 2: RWP 12%; RCT 0%), brain metastases (RWP 24%; RCT 0%), and life expectancy at start of treatment (RWP 12% died within 3.25 months of treatment; RCT excluded patients with <3 months life expectancy). 

## 4. Conclusions

Despite differences in patient characteristics such as ECOG performance status, brain metastases, and life expectancy at treatment initiation, PFS and OS at 6 months appear to be similar between the two cohorts. Further study with longer follow up and larger sample size is warranted to confirm these results. 

## 

*30_CAMO_2021*

**Differential Responses to a Carboplatin Containing Regimen in the Human Epidermal Growth Factor Receptor 2-Positive, Hormone Receptor-Negative Breast Cancer Patient Population**

## 

MontemurriVanessa^1^*HusseinAbdulkadir^2^GuptaRasna^1^KulkarniSwati^1^KayAmin^1^MathewsJohn^1^HammCaroline^1^1Schulich School of Medicine, Windsor, ON N9B 3P4, Canada2University of Windsor, Windsor, ON N9B 3P4, Canada*Correspondence: vmontemurri2023@meds.uwo.ca

 

## 1. Background

The HER2-positive, HR-negative breast cancer population is one that is widely unexplored. Studies have shown decreased toxicity benefits when using carboplatin based chemotherapy in HER2+ patients.

## 2. Objective

We set out to compare the outcomes of HER2+/HR− patients that received carboplatin (a non-anthracycline) containing therapy, to those that received the standard therapy which uses an anthracycline.

## 3. Methods

We explored our database of 1734 patients from 2004–2010 finding a total of 96 HER2+/HR− patients. Twenty patients were removed because they did not have invasive cancer or did not receive chemotherapy. A retrospective chart review was then conducted to compare progression free survival and overall survival in 76 HER2+/HR− patients, of which 62 received a non-carboplatin containing therapy and 14 received TCH (carboplatin containing).

## 4. Results

5.54% of patients in our database fit the phenotype of HER2+/HR−. Over a three year follow up, a progression free survival rate of 85.71% in the carboplatin group was found to be significantly higher than the 79.03% in the non-carboplatin group. An overall survival rate of 92.86% in the carboplatin group was also significantly higher than the 74.19% in the non-carboplatin group. The limitation here is the small sample size of the carboplatin group and shorter follow up time.

## 5. Conclusions

In conclusion, the HER2+/HR− phenotype is a small subset of patients, but we have shown that they have improved outcomes with carboplatin containing chemotherapy. The restrictions of this small retrospective study define this as hypothesis-generating only. A prospective study investigating this population of patients would help to define the best treatment for this unique group.

## 

*31_CAMO_2021*

**Study in Progress: Assessing the Impact of the COVID-19 Pandemic on Treatment Decision-Making and Care Experiences**

## 

AlsafarNoura^1^*LupichukSasha^1^,^2^AlimohamedNimira^1^,^2^SamnaniSunil^1^HaoDesiree^1^,^2^1Tom Baker Cancer Centre, Calgary, AB T2N 4N2, Canada2Cumming School of Medicine, University of Calgary, Calgary, AB T2N 1N4, Canada*Correspondence: noura.alsafar@albertahealthservices.ca

 

## 1. Background

To assess the impact of the COVID-19 pandemic on treatment decision-making and care experiences amongst cancer patients currently undergoing treatment in Alberta, Canada. We will also explore whether patient/disease factors such as curative intent vs. palliative intent treatment, duration of cancer treatments, adequacy of COVID-19 precautions, anxiety level, or testing positive for COVID-19 would influence reported responses. 

## 2. Methods

Based on the results of the literature review, existing instruments and iterative feedback from medical oncologists, nurses, and a patient volunteer, a 24 item patient survey was constructed by the study team. This survey includes items for measuring the increasing concern from the patients regarding their vulnerable health, canceled procedures and operations, possible cancellations, delays or adjustments to their treatment schedules, willingness to get the COVID-19 vaccine, and transition from face-to-face consultation to telemedicine. The aim is to accrue 100 patients starting 20 January 2021. For patients who have consented and completed the survey, chart review will be performed to collect accurate information about demographics, cancer diagnosis, and treatments. Descriptive statistics will be utilized to describe the cohort and survey responses. Logistic regression will be used to assess for variables associated with treatment-decisions potentially altered by the COVID-19 pandemic.

## 3. Results

Interim results describing summary of chart review and survey data will be available in April 2021 for presentation. 

## 4. Conclusions

To our knowledge, this will be the first study in Alberta to assess oncology patients’ behaviors and experiences relating to the ongoing COVID-19 pandemic, as well as the impact on the patient’s treatment seeking behavior. Our findings can be used to inform quality assurance and/or quality improvement strategies for patient populations during pandemic scenarios. Additionally, data from this study can also help inform whether measures undertaken during this outbreak, such as virtual care, can be leveraged as long-term solutions under regular operations.

